# Upon Certain Coincidences in Cases of Retained Placenta

**Published:** 1866-06

**Authors:** H. Webster Jones

**Affiliations:** One of the Physicians to Cook County Hospital


*Upon Certain Coincidences in Cases of Retained Placenta.* By H. WEBSTER JONES, A. M., M. D., one of the Physicians to Cook County Hospital.

Having noticed a singular agreement in the phenomena attending the last three cases of retained placenta, which have occurred under my own observation, I venture to place them upon record, with a view to determine from the results of comparative experience what relationship of cause to effect may have therein subsisted.

CASE I. Mrs. P., æt. 16, a primipara and healthy, was found upon her bed, having, a half an hour previously, while at the supper table, discharged the liquor amnii in great quantity. Her pains were slight and infrequent, and from the os uteri protruded a long loop of the funis. Early in the evening, the patient obstinately refused to assume the position recommended in such cases for the repositing of the cord, and other means proven unavailing, she was left to assume herself the responsibility of the infant's death. The labor was otherwise normal, though, throughout the whole, the uterine action was tardy and inefficient. Birth took place at 8 A. M., the lifeless infant exhibiting every indication of a vigorous intra-uterine growth.

The contraction of the womb was now cylindrical, persistent, but not firm, and the hemorrhage being so great as to demand other than ordinary remedies, I proceeded to extract the placenta manually at 8.45 A. M. It was found attached in the median line, anteriorly, extending directly upward from near the os to the fundus uteri, the attachment narrow in its whole extent, and requiring moderate effort to secure its severance. A firm globular contraction ensued, with immediate relief to the hemorrhage.

The afterbirth was oblong, resembling the battledore variety, and was not notably denser in texture than usual.

The patient recovered rapidly without an untoward symptom.

CASE II. Mrs. W., æt. 24, third pregnancy, was found having regular pains about the end of the sixth month. She had been feeble during the early months, but knew of no cause for untimely labor. Her pains were not severe, but at 5 P. M. she discharged the amniotic fluid in vast quantity, birth soon following of an ill-nourished child, which lived nearly twenty-four hours. Cylindrical uterine contraction followed, with no hemorrhage. Ergot given in gr. xx doses, once in twenty minutes, secured (after the third) violent action, without resulting in the extrusion of the placenta. Chloroform was given at 7 P. M., and after much difficulty, the placenta seized and delivered manually. Its shape, texture and place of attachment was identical with that before described.

The patient recovered slowly, with no unusual symptom, unless in the peculiar offensiveness of the lochia, which persisted for about four weeks.

This lady had attained the same period in her first pregnancy when she began to be troubled with discharges of a fluid per vaginam, which resembled, and probably was, the liquor amnii. They occurred always at night, often waking her from sound sleep, and resulted after three weeks in the birth of a fœtus, which had obviously deceased many days before. The placenta was so disintegrated as to prevent examination.

Her second pregnancy was normal in every respect, and the child perfect and healthy in an extraordinary degree.

CASE III. Mrs. S., æt. 39, second pregnancy, was in pains—slight and infrequent—for twelve hours before I saw her, at 5 A. M. Os dilated and membranes protruding. Liquor amnii was in great excess, and after its discharge, the contractions were more efficient. Birth took place at 9 A. M. Alarming hemorrhage following, and the uterus assuming the hour-glass form, I delivered the placenta at once by the usual operation.

Cold water, and ice externally and within the vagina, were necessary before sufficient contraction of the uterine fibre was induced, to secure the patient from further loss of blood. Even then the form of the womb was cylindrical, extending upward nearly to the umbilicus.

The location of the placenta was the same as in the other cases; it was very narrow in its line of attachment, and had been partially separated at the sides, where, evidently, the hemorrhage had originated. No evidences of inflammation were present, and with the exception of the tearing of the uterine surface in the process of removal, the appearance of the whole was normal.

This patient recovered rapidly, though suffering from chill and subsequent fever on the fifth and ninth days.

The peculiar interest of these cases rests in the common fact of a great excess in the quantity of liquor amnii; inertia of the uterus; retention of the placenta; the locality of its attachment, and finally, the normal condition of its texture.

My own impression of the relationship of these facts is as follows: That the great distention of the uterus, by reason of what might properly be called the "dropsy of the amnion," occasioned the uterine inertia; that the longitudinal muscular fibres, never very largely developed, were paralyzed, giving rise to the peculiar cylindrical form of the uterine contraction, and its inability to extrude the placenta; while the non-inflammatory nature of the placental attachment would tend to the belief that its retention was due to its peculiar shape and locality, in connection with inertia of the uterus, and loss of power in its longitudinal axis.

Practically, the concurrence of such phenomena as preceded birth in these cases should teach the accoucheur the presence of greater risk from hemorrhage, and the stronger probability of an operation being requisite for the delivery of the placenta.

